# 4421 Exposure to topical antimicrobials reduces inflammatory gene expression in keratinocytes

**DOI:** 10.1017/cts.2020.390

**Published:** 2020-07-29

**Authors:** Sirisha Sirobhushanam, Allison C Billi, Celine C Berthier, Lam C Tsoi, Johann E Gudjonsson, J Michelle Kahlenberg

**Affiliations:** 1 University of Michigan School of Medicine; 2 University of Michigan

## Abstract

OBJECTIVES/GOALS: Lupus lesional skin has elevated interferon expression, is highly colonized with *Staphylococcus aureus* (50%) and has no FDA-approved treatment options. We decided to investigate the effect of topical antibiotics on lupus lesional skin to determine whether it affects inflammatory gene expression. METHODS/STUDY POPULATION: Adult Systemic Lupus Erythematosus (SLE) patients with skin inflammation were recruited for this study from the Michigan Lupus cohort. All patients gave informed consent approved by the University of Michigan IRB. Lesions were swabbed for *S. aureus* colonization and then skin biopsies were collected from the affected area. Patients were then randomized for either mupirocin treatment or Vaseline^TM^ as the control. Product was applied to the lesion thrice daily for 7 days and swab samples and biopsies were collected again. Biopsies were saved at −80 °C. RNA was isolated from the biopsies, checked for quality and RNA-sequencing was performed to determine transcriptomic changes. RESULTS/ANTICIPATED RESULTS: Our preliminary results indicate that a higher number of genes are differentially expressed (DEGs) following treatment with mupirocin (184) than Vaseline^TM^ (133). Interestingly the DEGs from the two treatments were almost completely independent with only a few that were DE in both treatments when the data were fitted to a scatter plot. Functional enrichment analysis of the data showed significant downregulation of cytokine and chemokine pathways in the mupirocin but not the Vaseline^TM^ treatment group. DISCUSSION/SIGNIFICANCE OF IMPACT: Our preliminary data suggests that inflammatory signaling can be reduced in lesional skin by reducing bacterial load by topical antibiotic treatment in lupus patients. This can be particularly helpful in patients who are recalcitrant to typical treatment protocols for skin inflammation. CONFLICT OF INTEREST DESCRIPTION: J.M.K. received research funding from Celgene and serves on advisory boards for AstraZeneca, Boehringer Ingelheim, Bristol Myers Squibb, and Eli Lilly and J.E.G. received research funding from AbbVie, SunPharma, Celgene, and Genentech and serves on advisory boards for Novartis, AbbVie, and MiRagen. The other authors have no financial conflicts of interest.

